# Preparation of Synthetic Zeolites from Coal Fly Ash by Hydrothermal Synthesis

**DOI:** 10.3390/ma14051267

**Published:** 2021-03-07

**Authors:** David Längauer, Vladimír Čablík, Slavomír Hredzák, Anton Zubrik, Marek Matik, Zuzana Danková

**Affiliations:** 1 Department of Environmental Engineering, Faculty of Mining and Geology, VŠB—Technical University of Ostrava, 17 listopadu 15, 708 33 Ostrava-Poruba, Czech Republic; vladimir.cablik@vsb.cz; 2 Institute of Geotechnics of Slovak Academy of Sciences, Watsonova 45, 040 01 Košice, Slovakia; hredzak@saske.sk (S.H.); zubant@saske.sk (A.Z.); matik@saske.sk (M.M.); 3 State Geological Institute of Dionýz Štúr Bratislava, Regional Centre Košice, Jesenského 8, 040 01 Košice, Slovakia; zuzana.dankova@geology.sk

**Keywords:** coal fly ash, zeolite, hydrothermal synthesis, sodalite, surface, XRD, SEM

## Abstract

Large amounts of coal combustion products (as solid products of thermal power plants) with different chemical and physical properties cause serious environmental problems. Even though coal fly ash is a coal combustion product, it has a wide range of applications (e.g., in construction, metallurgy, chemical production, reclamation etc.). One of its potential uses is in zeolitization to obtain a higher added value of the product. The aim of this paper is to produce a material with sufficient textural properties used, for example, for environmental purposes (an adsorbent) and/or storage material. In practice, the coal fly ash (No. 1 and No. 2) from Czech power plants was firstly characterized in detail (X-ray diffraction (XRD), X-ray fluorescence (XRF), scanning electron microscopy with energy dispersive X-ray analysis (SEM-EDX), particle size measurement, and textural analysis), and then it was hydrothermally treated to synthetize zeolites. Different concentrations of NaOH, LiCl, Al_2_O_3_, and aqueous glass; different temperature effects (90–120 °C); and different process lengths (6–48 h) were studied. Furthermore, most of the experiments were supplemented with a crystallization phase that was run for 16 h at 50 °C. After qualitative product analysis (SEM-EDX, XRD, and textural analytics), quantitative XRD evaluation with an internal standard was used for zeolitization process evaluation. Sodalite (SOD), phillipsite (PHI), chabazite (CHA), faujasite-Na (FAU-Na), and faujasite-Ca (FAU-Ca) were obtained as the zeolite phases. The content of these zeolite phases ranged from 2.09 to 43.79%. The best conditions for the zeolite phase formation were as follows: 4 M NaOH, 4 mL 10% LiCl, liquid/solid ratio of 30:1, silica/alumina ratio change from 2:1 to 1:1, temperature of 120 °C, process time of 24 h, and a crystallization phase for 16 h at 50 °C.

## 1. Introduction

A total of 122 million tons (Mt) of coal combustion products (CCPs) is produced worldwide. From this amount, 40 Mt is produced in EU 15 (Austria, Belgium, Denmark, Finland, France, Germany, Greece, Ireland, Italy, Luxembourg, Netherlands, Portugal, Spain, Sweden, and the United Kingdom). Coal fly ash production in EU 15 is estimated at 25 Mt [1]. This amount is produced worldwide by thermal power plants. CCPs should be used as much as possible because the disposal of such a large amount of coal fly ash becomes a serious problem for the environment. The utilization rate depends on the region where CCPs are produced. The minimum utilization of coal fly ash is at the level of 10% in Africa and the Middle East and the maximum utilization is around 99% in Japan. The average utilization rate is about 64% worldwide [1,2].

Materials useful for zeolite synthesis can be silicon- and aluminum-rich chemicals, minerals available in the earth’s crust or industrial waste (blast furnace slag [3], aluminum dross, Liquid Crystal Display (LCD) glass [4], coal shale [5], etc.). Coal fly ash is a cheap and abundant product, rich in minerals containing silicon and aluminum, making it suitable as a starting material for the synthesis of zeolites. The types of zeolites that are formed by hydrothermal synthesis depend on the influence of temperature, pressure, the concentration of chemicals used in the process, pH, activation time, and the ratio of SiO_2_ and Al_2_O_3_ in the input material [6]. Suitable coal fly ash for this method appears to be coal fly ash from a high-temperature process as a starting material for the synthesis of zeolites. The raw material is incinerated at a temperature of 1200–1700 °C. The most represented here is molten silica in the form of spherical glass particles; the content is about 50%. It is a very fine ash that has pozzolanic activity. Ash formed by fluidized bed combustion appears to be unsatisfactory for synthesis; the combustion temperature is 800–850 °C. This ash contains unreacted CaO (15–35%), which is reactive, and it has a relatively high SO_3_ content of 7–13%. Due to its composition, it is not suitable for the synthesis of zeolites [7]. Zeolites were first identified by Cronsted in 1756, but their molecular and structural properties remained unknown until 1920. Their consists of a crystalline structure built from [AlO_4_]^5−^ and [SiO_4_]^4−^ bonded together by four oxygen atoms located in the corners of a tetrahedron, which are shared with other tetrahedra. Natural zeolites can be formed in small cavities of basaltic rock over the years or as volcanic tuffs or glass altered by interaction with saline water. They can be found in alkaline deserts, lake sediments, ash ponds, marine sediment, or metamorphic rocks [6]. The general formula of natural zeolites is shown in Equation (1). Synthetic zeolites are synthesized by chemical processes. The materials useful for those processes are chemicals, minerals, or industrial products that are rich in silica and alumina content. Synthesized zeolites usually have better chemical and physical properties than nature zeolites (bulk density, specific gravity, porosity, cation exchange capacity, specific surface area, pore radius, particle size, adsorption, etc.) [8].

(1) $${\left(Li,\;Na,\;K\right)}_{p}{\left(Mg,\;Ca\;,Sr,\;Ba\right)}_{q}\left[Al_{\left(p+2q)\right)}Si_{n-\left(p+2q\right)}O_{2n}\right]\times m_{o}H_{2}O$$

Various methods for preparing synthetic zeolites are described in articles [6,8,9,10,11,12,13,14,15,16,17,18,19,20,21,22,23,24,25,26,27,28,29]. The main methods of preparation include the convection hydrothermal method, the hydrothermal method using microwave waves, the salt melting method and the hydrothermal fusion method [6]. These processes use different types of chemicals, process times, and temperatures, and different types of zeolites are also created, as tabulated in Table 1.

The method of conventional hydrothermal synthesis was used in this work. In works when this method was used, the yield of the zeolite phase was about 20–60 wt.% [9,10,22,23,24,25,26,27]. However, it always depends on the type and properties of the coal fly ash. The amount of zeolite phases formed is influenced by parameters such as type and composition of the coal fly ash, concentration and type of chemicals (NaOH, KOH, LiOH, H_2_SO_4_, HCl), temperature, process time, Si/Al, and liquid/solid (L/S) ratio [30].

Conventional hydrothermal synthesis for the production of zeolites from coal fly ashes was studied by many authors (the zeolite phase varied in the range 20–60 wt.%) [9,10,22,23,24,25,26,27]. However, the study on the influence of the combination of several conditions, such as process length, temperature effect, and the effects of the chemical treatment (NaOH, LiCl, Al_2_O_3_, aqueous glass), is missing.

LiCl was used in this work because it is a source of Li^+^ and Cl^−^. Lithium has the same function in processes as Na^+^ or K^+^ ions do, depending on which hydroxide is used for the process of hydrothermal synthesis. Cl^−^ anions have a strong structure-directing effect on the sodalite framework structure and on the formation of sodalite. Thus, the main goal of the work is to find the parameters for the hydrothermal zeolitization process. Selected products with a high content of the zeolite phase were characterized from the viewpoint of texture properties, morphology, and phase composition.

## 2. Materials and Methods

### 2.1. Coal Fly Ashes for Zeolitization

Coal fly ash (CFA) used for hydrothermal synthesis was obtained from the Dětmarovice power plant (the Czech Republic). The Dětmarovice power plant burns black coal with an average calorific value of 22 MJ/kg and a sulfur content below 0.5%. The power plant works with 4 power units each with an electrical output of 200 MW. The average daily fuel consumption per unit is about 1600 t of coal. After being ground into a very fine fraction, the coal is burned in a total of 4 boilers with an output of 650 t of steam an hour. The boilers are pressure, two-pass boilers, with a granulation combustion chamber. Their efficiency is around 90%, and the highest temperature in the boiler is 1400 °C [31].

Two coal fly ash samples were used in this study, namely, coal fly ash No. 1, which was from electrostatic precipitators, and coal fly ash No. 2, which was from a mechanical stage. Coal fly ashes No. 1 and No. 2 were subjected to input analyses (particle size measurement, specific surface area, XRD, X-ray fluorescence (XRF), SEM, energy dispersive X-ray analysis (EDX), and loss on ignition (LOI)). Using this analysis, basic information about these input materials for the process of hydrothermal synthesis was obtained.

### 2.2. Analysis of Initial Samples and Synthesized Products

Particle size analysis was performed by CILAS 1190 (Cilas Arianegoup, Orleans, France). It used a wet method in an aqueous/alcohols medium. The measurement length of one sample was 60 s, and the measurement range was from 0.04 to 2500.00 µm.

A fluorescence, X-ray spectrometer Spectroscan Makc GVII (Spectron Optel, Wed Petersburg, Russia) was used for the samples of coal fly ash to determine the chemical composition.

A Bruker Advance D8 instrument (Bruker AXS, Karlsruhe, Germany) was used for the sample of coal fly ash and zeolite phases to determine the mineralogical composition. The samples were measured in the laboratories of VŠB-TUO (VSB—Technical University of Ostrava, Ostrava, the Czech Republic) and The Institute of Geotechnics, The Slovak Academy of Sciences, Košice, Slovakia. Both workplaces have the same type of device. The measurement conditions were as follows: start: 5°, end: 80°, step: 0.04°, step time: 20 s, temperature: 25 °C. Zinc oxide was added to the samples in order to precisely determine the content of the amorphous phase (according to the Czech standard ČSN 650102 from 1979, we used ZnO (f.a.) for analysis 99–99.8% purity). Diffracplus Topas software (Version 4.2, Bruker AXS, MA, USA) was applied for quantitative data evaluation.

Particle morphology was studied by field-emission scanning electron microscopy with an energy dispersive FEI Quanta 650 FEG X-ray (ThermoFisher Scientific, OR, USA).

The surface properties of the samples were determined from the adsorption isotherms measured using a NOVA 1200e Surface Area & Pore Size Analyzer (Quantachrome Instruments, Boynton Beach, FL, USA) by the physical adsorption of nitrogen at −196 °C. First, the samples were degassed at 150 °C in a vacuum oven under a pressure lower than 2 Pa for 18 h. The measured data were processed by the Brunnauer–Emmett–Teller (BET) isotherm in relative pressure with a range of 0.05–0.3 to obtain the value of the specific surface area (S_BET_) [32]. The values of the external surface (S_t_) and volume of micropores (V_micro_) were calculated from the t-plot using the Harkins–Jura standard isotherm [33]. For the input samples and sample 6B, the whole adsorption and desorption isotherms were measured under the same conditions. The curves of adsorption studies and isotherms were prepared using Sigma plot (Version 12), scientific data analysis, and graphing software.

### 2.3. Hydrothermal Method

**Figure 1:**
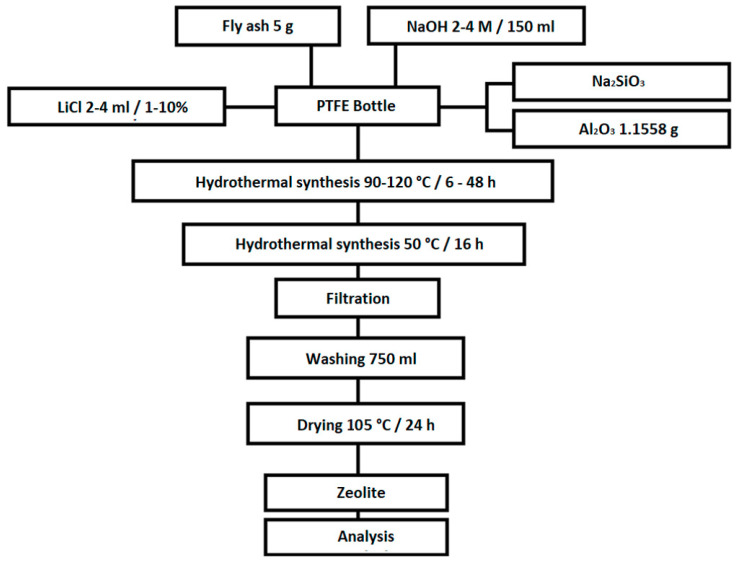
Scheme of the experiments.

At the beginning of each experiment, the coal fly ash was weighed on analytical scales. The amount of the coal fly ash was 20 g in the initial experiments (0, 0A, 0B, 0C, 0D, and 0E). In all other experiments, the amount of coal fly ash was 5 g. The coal fly ash was transferred to a 250 mL Teflon bottle. Then, 150 mL NaOH was added to this amount of coal fly ash at various concentrations of 2–4 M. The liquid/solid (L/S) ratio was 7.5:1 or 30:1. In the literature, the authors list various suitable L/S ratios, which range from 2:1 to 250:1 [16,27,28]. In the initial experiments for the given conditions, this part was final and the closed Teflon vessel was placed in an oven at temperatures from 90 to 120 °C, for a period of from 6 to 48 h. After this phase, the sample was placed in another oven at 50 °C for 16 h, at which time the hydrothermal synthesis continued at reduced temperatures. Samples labelled 0, 0A, 0B, 0C, 0D, and 0E were not subjected to this phase. The previous part of the procedure was the same for all experiments presented in this work. The following steps are slightly different for different types of experiments. In the selected experiments, different amounts and concentrations of the LiCl solution, Al_2_O_3_, or aqueous glass (as a source of SiO_2_) were added. The experiments were conducted with a Si/Al ratio of 2:1 (except of samples 1C and 1D—the Si/Al ratio was 3:1 and samples 5, 5A, 5B, 6, 6A, and 6B, where the Si/Al ratio was 1:1). Exact data on the amount and concentrations of chemicals, process lengths, and temperature are shown in Chapter 3.2. At the end of the experiments, the contents of the Teflon bottle were filtered with 750 mL of demi-water to lower the pH (pH~8). This step resulted in washing out the excess NaOH and also stopping the zeolitization process. The washed sample was placed in an oven and dried at 105 °C for 24 h. After drying, the samples were subjected to XRD, SEM, and specific surface area analyses. The scheme of the experiments is shown on Figure 1.

## 3. Results

### 3.1. Characterization of Initial Material (CFA)

**Figure 2:**
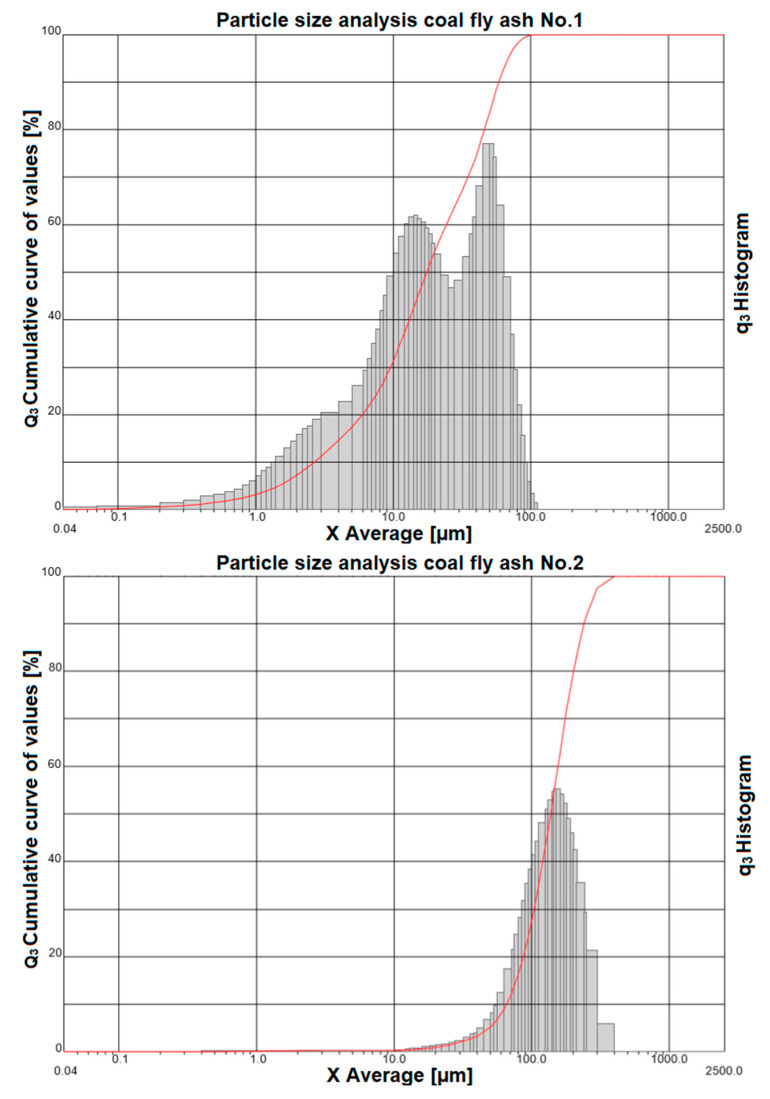
Particle size analysis of coal fly ash No. 1 (top image) and coal fly ash No. 2 (bottom image).

The results of the granulometric analysis us that coal fly ash No. 1 is composed of particles with a size from 0.04 µm to particles with a size of 112 µm. Figure 2 (top image) shows the representation of individual grain classes using a histogram. The cumulative curve shows that the particle diameter at 10% is 2.66 µm, the diameter at 50% is 17.52 µm, the diameter at 90% is 58.59 µm, and the average diameter of the whole sample is 25.38 µm.

The granulometric analysis shows that coal fly ash No. 2 is composed of particles with a size from 0.5 µm to particles with a size of 400 µm. Figure 2 (bottom image) shows the representation of individual grain classes using a histogram. The same figure also shows a cumulative curve, where the particle diameter at 10% is 65.52 µm, the diameter at 50% is 137.42 µm, the diameter at 90% is 240.92 µm, and the average diameter of the whole sample is 146.8 µm.

The XRF results show the chemical compositions of coal fly ashes No. 1 and No. 2, which are tabulated in Table 2. The major compounds in both coal fly ash samples include SiO_2_, Al_2_O_3_, Fe_2_O_3_, CaO, K_2_O, and MgO. Minor compounds include Na_2_O, P_2_O_5_, SO_3_, TiO_2_, and MnO.

**Figure 3:**
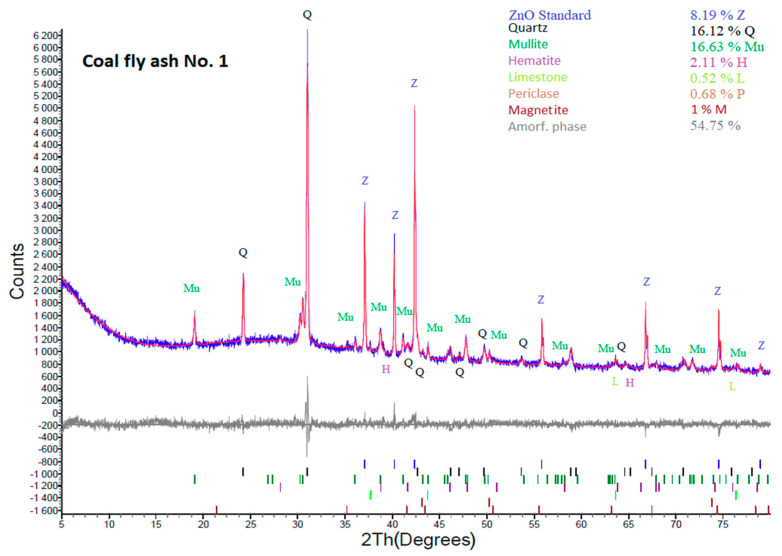
XRD analysis of coal fly ash No. 1.

**Figure 4:**
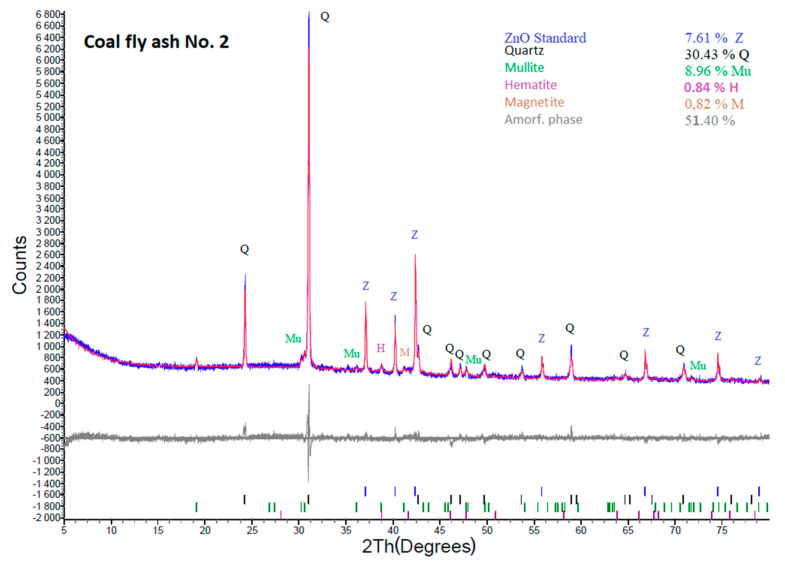
XRD analysis of coal fly ash No. 2.

According to the XRD results (Figure 3 and Figure 4), both coal fly ash samples contain hematite and magnetite. Fe_2_O_3_ was also confirmed by the semi-quantitative XRF. The presence of Fe_2_O_3_ causes lower yields of the zeolite phase in the hydrothermal synthesis process. In some cases, iron oxides are removed by magnetic separators or by leaching in acids [22,34]. In this work, these components were not removed from the coal fly ash. It was decided to keep Fe_2_O_3_ in the coal fly ash and see what amount of the zeolite phase could be created by the different process conditions with a higher amount of Fe_2_O_3_. Of course, the removal in this phase could have a positive effect on the content of the zeolite phase. Such experiments will be the next step of our research.

Except qualitative XRD data inspection, the obtained diffractograms (Figure 3 and Figure 4) were quantitatively evaluated, as shown in Table 3. The percentages of individual mineral phases with the content of the ZnO internal standard and after recalculation without ZnO content are tabulated. XRD results show that the amorphous phase in coal fly ash No. 1 is 59.63%. The sample also contains quartz (SiO_2_; 17.56%), mullite (3Al_2_O_3_·2SiO_2_; 18.11%), hematite (Fe_2_O_3_; 2.3%), magnetite (Fe_3_O_4_; 1.09), limestone (CaCO_3_; 0.57%), and periclase (MgO; 0.74%).

In comparison with sample No. 1, coal fly ash No. 2 contains a lower content of the amorphous phase (55.63%). It also contains quartz (32.94%), mullite (9.7%), hematite (0.84%), and magnetite (0.89%). Notably, the mineral phase composition of the studied coal fly ash together with the amorphous phase content greatly influences the hydrothermally induced zeolitization process (as is shown later).

**Figure 5:**
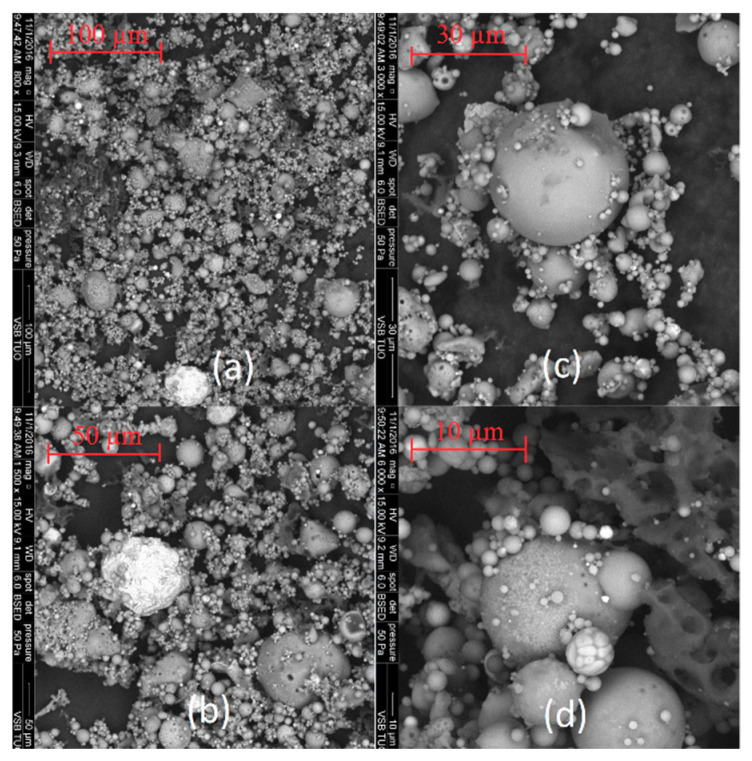
Coal fly ash No. 1 (**a**–**d**)—left side.

**Figure 6:**
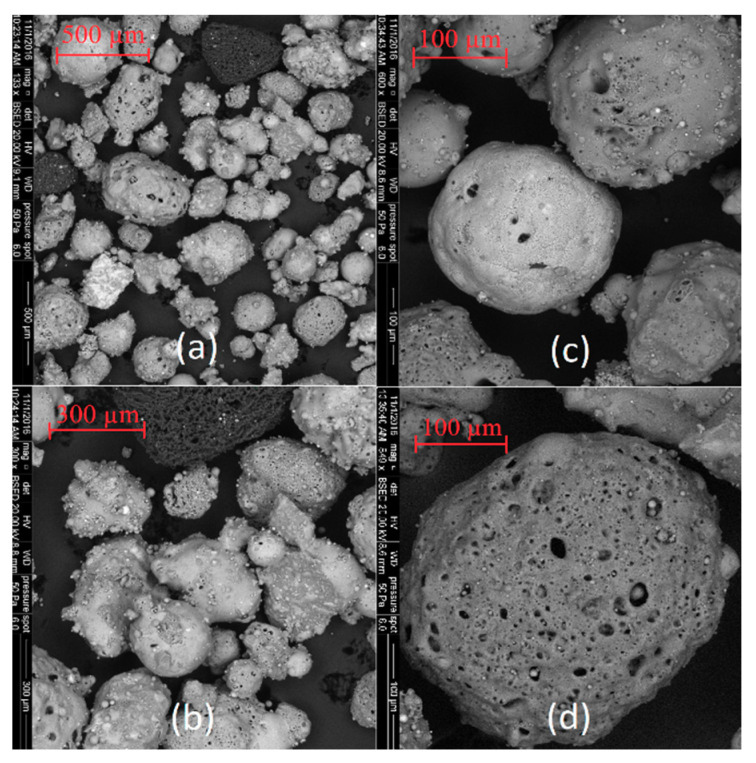
Coal fly ash No. 2 (**a**–**d**)—right side.

The SEM images of coal fly ashes No. 1 and No. 2. in various approximations are showed in Figure 5 and Figure 6. Figure 5 shows SEM images of coal fly ash No. 1, and Figure 6 shows SEM images of coal fly ash No. 2. Spherical particles can be seen. The spherical shape of the particles was formed during the high-temperature combustion process (1400 °C) of black coal in the power plant. The results from the particle size analysis (Figure 2) were confirmed. Sample No. 1 is finer compared to coal fly ash No. 2 (see Figure 5a and Figure 6c,d).

**Figure 7:**
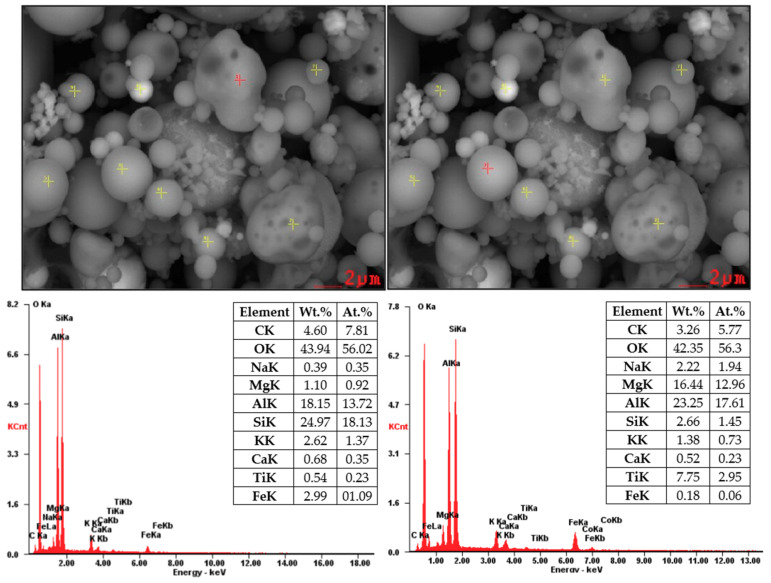
SEM-EDX analysis of coal fly ash No. 1 (local analysis—red points).

**Figure 8:**
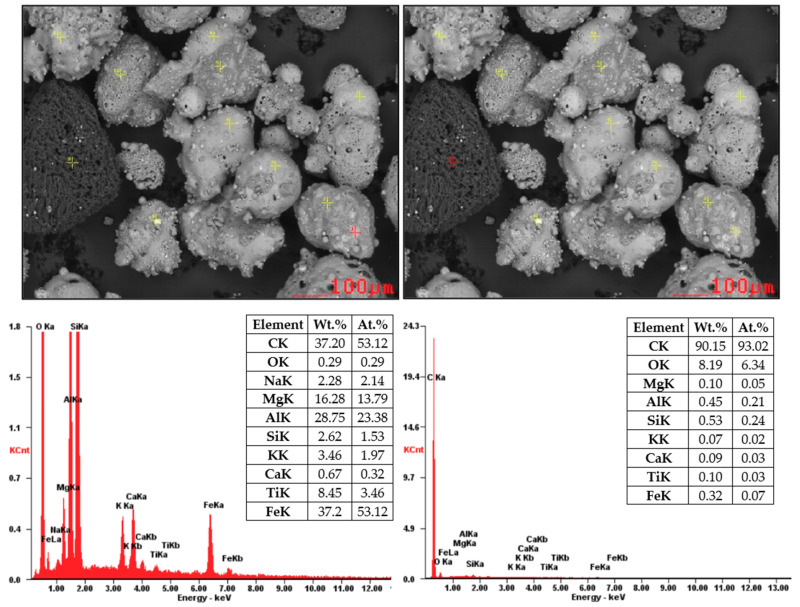
SEM-EDX analysis of coal fly ash No. 2 (local analysis—red points).

Figure 7 and Figure 8 showed SEM images supplemented by EDX analysis. These particles are composed mainly from O_2_, Al, and Si (aluminosilicates) containing Mg, K, Ca, and Fe. Non-burned particles were also monitored in the sample of coal fly ash No. 2. Figure 8 shows the results of EDX analysis of the non-burned particles, which consist mainly of carbon.

**Figure 9:**
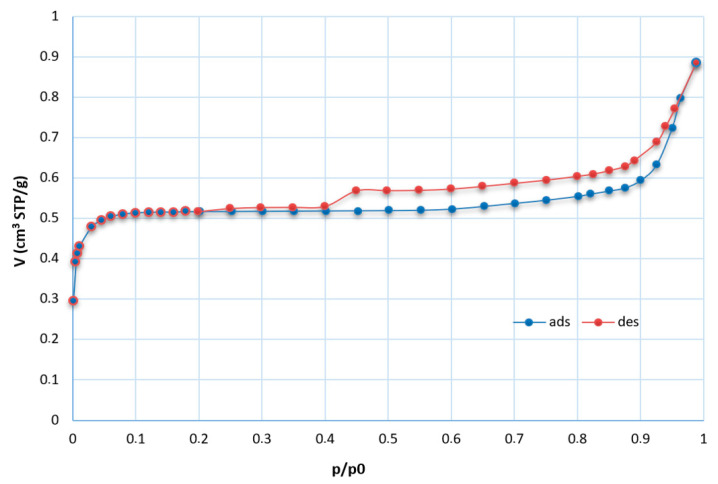
Isotherms for coal fly ash No. 1 (STP—standard temperature and pressure, t = 0 °C, *p* = 101.325 kPa; ads—adsorption isotherm (blue); des—desorption isotherm (red)).

The textural properties of the input samples were determined from the measured adsorption/desorption isotherms, and the values of specific surface area and external surface were calculated. The results of these analyses show S_BET_ values of 0.8 and 3.6 m^2^/g for coal fly ashes No. 1 and No. 2, respectively. Coal fly ash No. 1 was used in most of these experiments. Therefore, the whole adsorption and desorption isotherms were measured (Figure 9) and analyzed for this sample. The first part of the obtained adsorption isotherm corresponds to type I according to the IUPAC report characteristic for the microporous materials [35]. The adsorbed gas increased rapidly at low relative pressure. The inner part of the isotherm showed a hysteresis loop connected with capillary condensation in the mesopores. The parallel branches of the adsorption and desorption isotherm in the wide range of relative pressure (constant adsorption/desorption) correspond to isotherm type IV and hysteresis loop type H4 (typical for activated carbons and some other nanoporous adsorbents). These isotherms are of a composite nature. The initial region of the reversible micropore filling is followed by multilayer physisorption and capillary condensation [36]. The final part represents modified type II (typical for multi-layered adsorption in macropores or for adsorption on the external surface in the interparticle space of particles of submicron size); the main ratio of adsorbed gas can be observed in the range of relative pressure *p*/*p*0 > 0.9. The adsorption isotherm is of unlimited adsorption character.

For both input samples, low values of specific surface area were calculated. Despite the very low value of the specific value of sample No. 1, its BET isotherm was not of a nonstandard type. The negative value of the C constant (intercept) was obtained. In this case, the BET isotherm is not of the physical meaning, and other analyses suitable for microporous materials should be applied. The input sample was the only comparable sample, and our laboratory is not equipped with the necessary analyzers. Therefore, in the case of negative C values of the products, the t-plot analyses of the isotherm were realized to also verify the microporous character of the samples.

### 3.2. Characterization of Synthetized Products by Textural Analysis, XRD, and SEM-EDX

**Figure 10:**
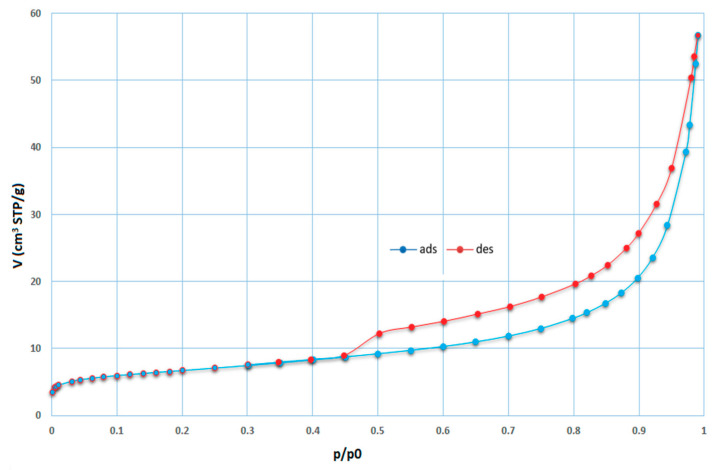
Adsorption (blue) and desorption (red) isotherms for sample 6B (STP—standard temperature and pressure, t = 0 °C, *p* = 101.325 kPa).

Several types of zeolite were formed during these experiments. Sodalite deserves special attention because if sodalite is prepared by hydrothermal synthesis, this sodalite is classified in the literature as a synthetic zeolite [6,9,10,37,38,39]. Sodalite is a rock-forming mineral with the general formula Na_8_Al_6_Si_6_O_24_·(X) with X = Cl^−^, CO_3_^2−^, SO_4_^2−^, OH^−^. Sodalite was formed in almost all of the experiments (in some cases, the zeolite phase was formed). Sodalite was always created in the majority phase of zeolite content in the synthetized product. The measured isotherms of sample 6B after the synthesis process are illustrated in Figure 10. For this sample, the S_BET_ value of 23.27 m^2^/g. was calculated, which is 30 times higher than that for input coal fly ash No. 1 (Figure 9). Sample 6B contains residuum and the zeolite phase created during synthesis. Therefore, S_BET_ is common to the overall phase. The shape of the adsorption–desorption isotherm can be classified as pseudo type II with a hysteresis loop type H3 (slip shaped pores). The final part of the isotherm, as in the case of the input sample, has an unlimited adsorption character.

The obtained results from the textural analyses (S_BET_, V_micro_, S_t_ values) for the selected products, as well as from the quantitative XRD analysis, are summarized in Table 4. The calculated values of the specific surface area were higher than for the input sample. For products 0C, 0E, 1, 1A, 1B, 1C, and 5A, they are marked in red. In these cases, negative C values were obtained. The values of the specific surface area of these samples are not of any physical meaning. For this reason, the t-plot method was applied on the adsorption data to verify their microporous character. They showed higher values of V_micro_ than other samples studied. The product samples of higher micropores volume contained, according to the XRD analyses, lower content (0.68–10.46%) of sodalite with Na-faujasite (FAU; Ca-FAU) zeolite (0.18–1.41%). For the other products (without the Na and Ca-FAU zeolite content), the microporous character was not so expressive.

A first, the non-zeolite phase in samples 0B (coal fly ash No. 2; 2 M NaOH) and 0D (coal fly ash No. 2; 3 M NaOH) was determined by quantitative XRD analysis. This was due to a high content of the Fe_2_O_3_ phase (10.6%) present in coal fly ash No. 2 (see Table 2). On the other hand, in the same conditions, samples 0C and 0E (coal fly ash No. 1; 2 M and 3 M NaOH), respectively, showed 4.43% (0C) and 5.12% (0E) of the zeolite phase (coal fly ash No. 1 contained 7.9% of Fe_2_O_3_ according to XRF). Therefore, coal fly ash No. 1 was used in the other experiments.

Selected diffractograms (highly relevant from our viewpoint) are shown (sample No. 4A; 6 and 6B). Table 5 shows the result of quantitative XRD analysis of the first row, even with the added ZnO standard, and of the second row. The results were recalculated without the ZnO standard, and the last row in this table shows the input values of coal fly ash recalculated without the ZnO standard. For the input conditions listed in the last row, the minor minerals contained in the coal fly ash were omitted if they were not already present in the hydrothermal synthesis product.

The effect of LiCl as well as Al_2_O_3_ from the viewpoint of different zeolite phase formations was clearly visible in samples No. 4A 6 and 6B. These samples were chosen for XRD profile determination. The main differences between the studied samples are that sample No. 4A was treated with low a concentration of LiCl (2 mL, 1%) for 48 h; sample No. 6 was treated for 24 h without any LiCl reagent, but Al_2_O_3_ was added to the reaction mixture; sample No. 6B contained both Al_2_O_3_ and LiCl (higher concentration, 4 mL of 10% LiCl), and the treatment time was 24 h. All other parameters were the same (temperature (120 °C), crystallization, L/S ratio, amount of NaOH). Thus, sample No. 6B exhibited the highest content of the sodalite phase without other additional components. Although sample 4A was treated for 48 h, a lower concentration of the zeolite phase was generated. This means that the addition LiCl and Al_2_O_3_ was a key parameter for the production of zeolite phases.

**Figure 11:**
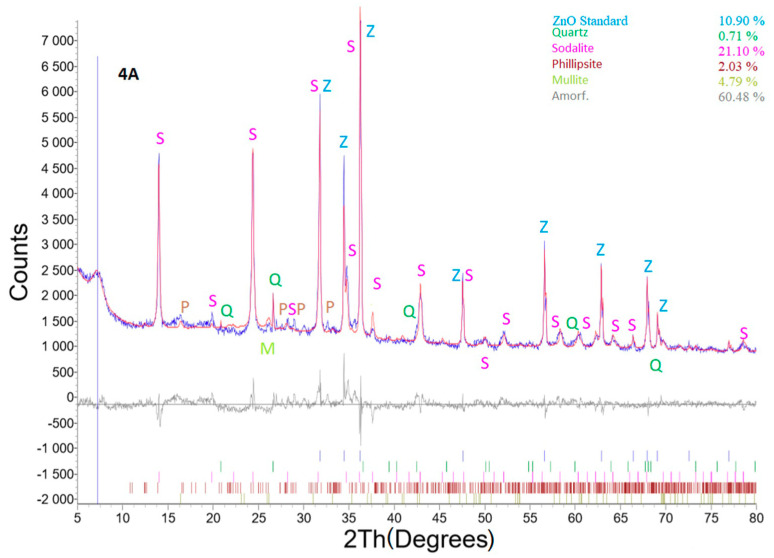
X-ray powder diffraction of sample No. 4A.

Figure 11 shows an XRD profile of sample No. 4A with the ZnO internal standard. The experimental hydrothermal process conditions were as follows: weight of coal fly ash No. 1—5 g, L/S ratio of 30:1, NaOH concentration of 4 M, temperature of 120 °C, catalyst addition of 2 mL 1% LiCl, process residence time of 48 h, and crystallization phase for 16 h at 50 °C. The hydrothermal treatment caused mineral phase transformations. Quartz and the mullite phase present in the coal fly ash sample changed to a zeolite structure (concretely sodalite—23.68% and phillipsite ((Ca,K,Na)_1-2_(Si,Al)_8_O_16_·6(H_2_O)—2.28%, see Table 5) [6,30]. The amount of mullite decreased from 18.11 to 5.38%. A higher decrease was monitored in the case of quartz (from 17.76 to 0.8%).

**Figure 12:**
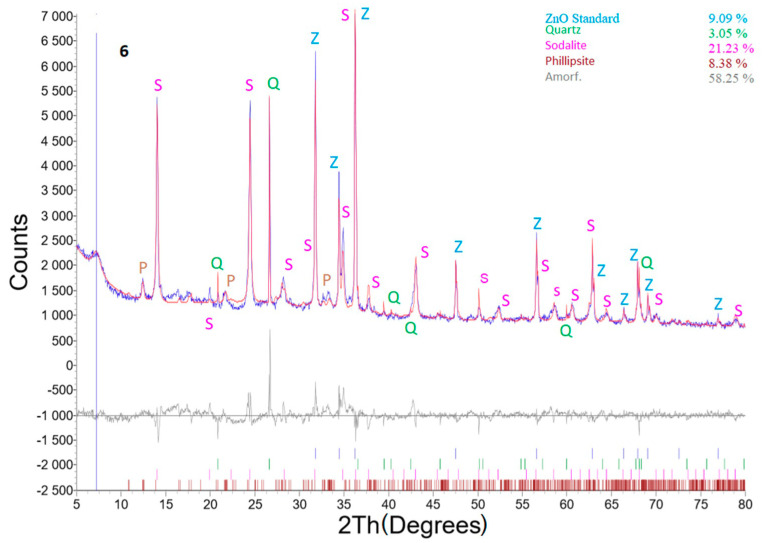
X-ray powder diffraction of sample No. 6.

Better results from the zeolitization process were obtained for sample No. 6 (Figure 12), where the process conditions were as follows: weight of coal fly ash No. 1—5 g, L/S ratio of 30:1, NaOH concentration of 4 M, temperature of 120 °C, addition of Al_2_O_3_—1.1558 g, residence time in this process of 24 h, and crystallization phase for 16 h at 50 °C. Interestingly, a non-mullite phase was recorded. Zeolite phase enrichment (sodalite—23.35%; phillipsite—9.22%) was observed (Table 5). Thus, the addition of Al_2_O_3_, caused a transformation connected with a higher formation of zeolite (in comparison to sample No. 4A) in the total mullite phase.

**Figure 13:**
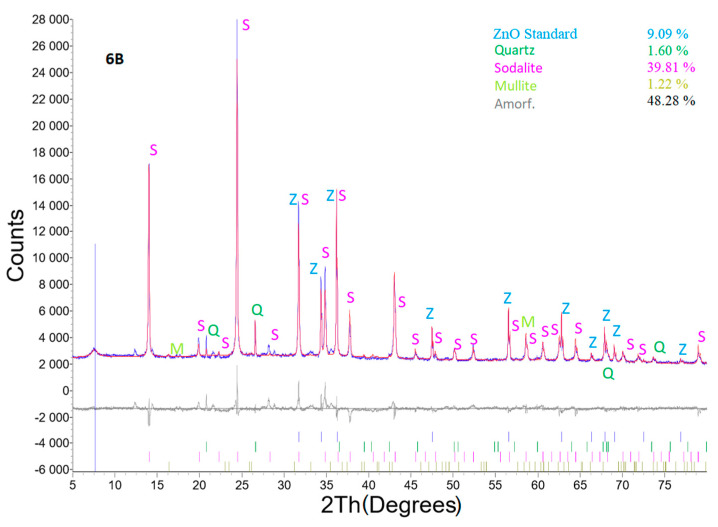
X-ray powder diffraction of sample No. 6B.

Figure 13 shows X-ray powder diffraction of sample No. 6B, where the best results were reached. The experimental conditions were as follows: coal fly ash No. 1—5 g, L/S ratio of 30:1, 4 M NaOH, temperature of 120 °C, 4 mL 10% LiCl, addition of Al_2_O_3_—1.1558 g, time of 24 h, and crystallization phase for 16 h at 50 °C. There was only one zeolite phase identified (sodalite), but with the highest content of 43.79%. Quartz and the mullite mineral phase decreased to a value of 1.76 and 1.34%, respectively.

**Figure 14:**
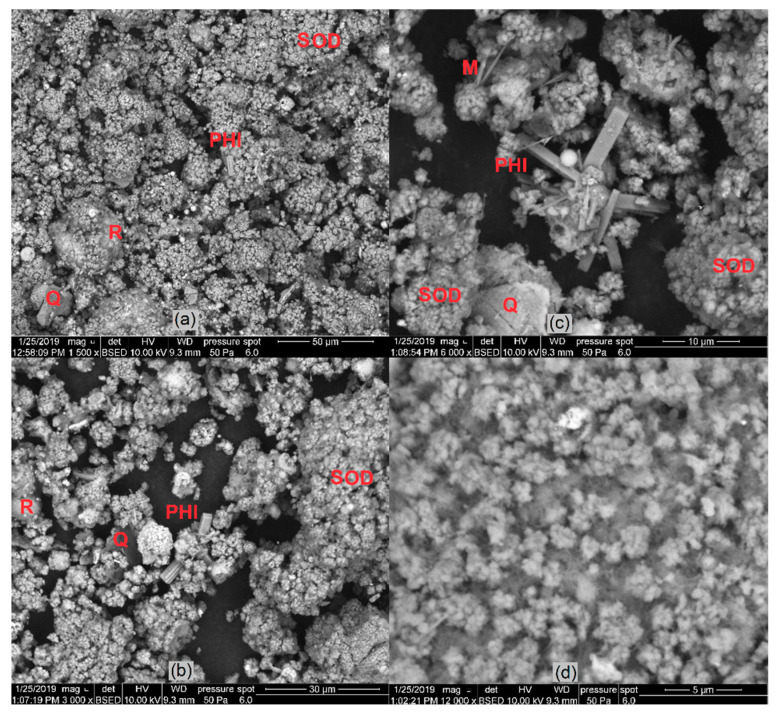
Images of zeolite phase formed by hydrothermal synthesis process: (**a**) sample No. 6 (sodalite, phillipsite) (**b**,**c**); sample No. 6A (sodalite, phillipsite); (**d**) sample No. 6B (sodalite).

The products of hydrothermal synthesis with the highest content of zeolite phases (samples No. 6, 6A, and 6B) were examined with SEM (Figure 14). Zeolite phases were identified from a morphology point of view, and the results of XRD analysis were confirmed. The spherical particles from input sample No. 1 were totally or partially dissolved and transformed into zeolite phases. Some of them were formed on the residuum surface. Mineral phases were labelled with symbols (SOD—sodalite, PHI—phillipsite, Q—quartz, M—Mullite, R—residuum) for better orientation—see Figure 14.

**Figure 15:**
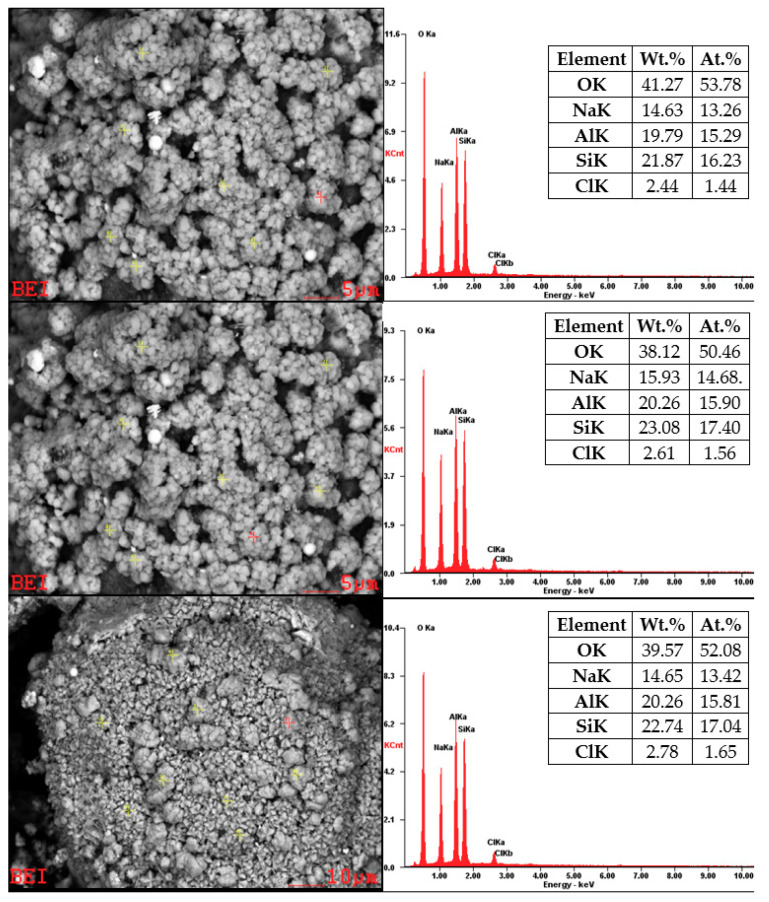
SEM-EDX for sample No. 6B.

Chemical analyses for the product of hydrothermal synthesis of sample No. 6B are shown in Figure 15. First, it is important to note that EDX analysis for particles smaller than 5 µm is only approximate measurement, because this technique cannot focus with accuracy directly on these mineral phases. The product obtained from the process is not a homogenous, and it contains sodalite (size of 0.5–1 µm) and residuum. The sodalite chemical composition is Na_8_Al_6_Si_6_O_24_·(X), where (X) = Cl^−^, CO_3_^2−^, SO_4_^2−^, OH^−^. Chemical analysis revealed that the content of all elements is identical to the composition of the chemical formula of sodalite, except Cl^−^. An explanation for these results in our opinion is that our chemical analysis showed us not only the chemical composition of sodalite alone but also the chemical composition of sodalite together with residuum (amorphous phase). This unidentified amorphous phase could be incompletely dissolved mineral phases from fly ash or an incompletely formed mineral phase of sodalite. Chlorine deficiency can also be caused by the fact that not only sodalite but also hydroxy-sodalite was formed during the process.

## 4. Discussion

### 4.1. Influence of the Parameters on the Process of Hydrothermal Synthesis

Various zeolite phases were prepared using the convection hydrothermal method. These were sodalite, phillipsite, Chabazite, faujasite-Na, and faujasite-Ca. Different conditions (temperature, time, NaOH concentration, LiCl concentration, Si/Al ratio, and L/S ratio) during the hydrothermal process causes different zeolite phase formations from both the qualitative and quantitative perspective. The conditions were changed and modified step by step, systemically, based on information obtained from previous experiments as well as from articles published previously by other scientists who have studied the zeolitization of coal fly ashes [6,9,10,11,12,13,14,15,16,17,18,19,20,21,22,23,24,25,26,27,28].

#### 4.1.1. Initial Sample

**Scheme 1:**
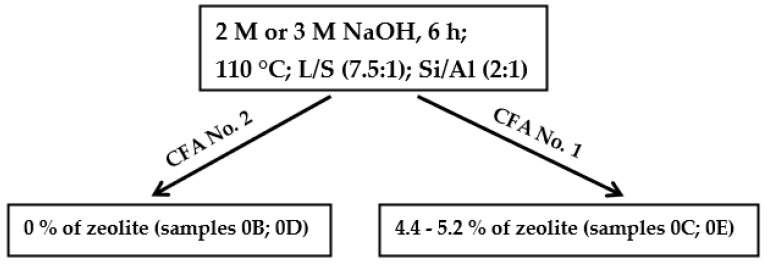
Experimental conditions in the initial experiments of hydrothermal synthesis.

In most experiments, coal fly ash No. 1 was used. This coal fly ash had better properties for hydrothermal synthesis than coal fly ash No. 2. The main differences were between the size of the particles (Figure 2 (left and right side), texture, and mineral phase composition (Table 2 and Table 3). According to semi-quantitative XRF analysis, coal fly ash No. 2 contained a higher amount of Fe_2_O_3_, CaO, and other impurities, which can have a negative influence on the process. Texture and granularity also played an important role. For the hydrothermal process, and for the contact between the solution and sample, it is important to dissolve the sample and to make contact with Na^+^ ions. Moreover, the first experiments showed that the zeolitization process is better with coal fly ash No. 1 (see Table 4, samples 0B, 0C, 0D, and 0E—the same experimental conditions for both coal fly ashes). In both cases, during the application of either 2 M or 3 M NaOH on coal fly ash No. 1, the zeolite phases (chabazite and faujasite-Na) were formed (up to 5.2%), whereas sample No. 2 was inactive in terms of zeolite formation (Scheme 1).

#### 4.1.2. Effect of Time, Temperature, and LiCl

**Scheme 2:**
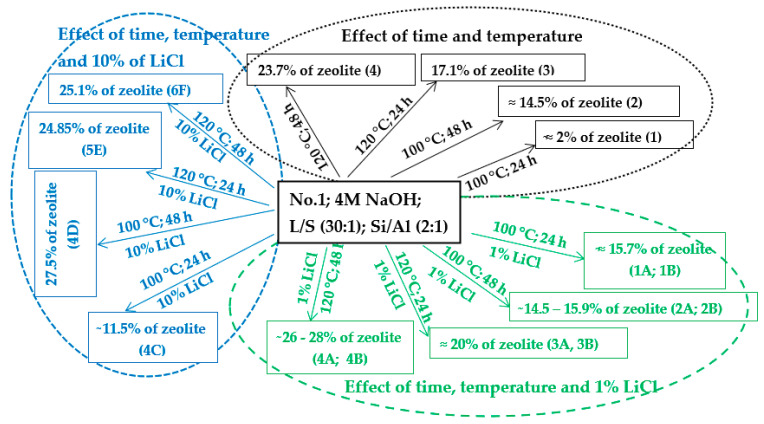
Hydrothermal synthesis of zeolites with and without the addition of LiCl (1 and 10%).

The variable in this work was time. Different authors give different views on the length of the process of hydrothermal synthesis [9,10,14,17,22,26,28,29,34,40,41,42]. Some authors state that a process length of 6 h without the crystallization phase is sufficient for the formation of zeolite phases in their experiments [10,26]. Some state that the ideal process length is 24 h, followed by a crystallization phase, at temperatures ranging from 30 to 90 °C [6,9]. Based on the results of the experiments in this work, where temperature and time were the only variables (Scheme 2—black part), it was concluded that extending the process time in the experiment from 24 to 48 h led to an increase in the zeolite phase over time at 100 and 120 °C. However, an extension of time from 24 to 48 h at 120 °C did not cause such a significant increase in the zeolite phase as at 100 °C (samples No. 1, 2, 3, and 4).

Different results were obtained in experiments where temperature and time were not the only variables. Thus, a different amount and concentration of LiCl solution were added to the individual experiments. The effect of LiCl was studied at two different temperatures (100 and 120 °C), at two different time periods (24 and 48 h), and various concentrations (1 and 10%)/volumes (2 and 4 mL). Scheme 2 (green part) shows the effect of time and temperature with the addition of 1% LiCl. The results of the experiments at a temperature of 100 °C and a process time of 24 h (1A and 1B) show that the content of the zeolite phase is much higher (≈15.6%) than that in an experiment with the same conditions but without 1% LiCl (0Aa ≈ 2%). In the experiment under the same conditions as in the previous experiments (1A and 1B) but with a longer process (48 h; 2A, 2B), unexpectedly, a higher amount of the zeolite phase was not obtained compared with the 24 h processes. The amount of the zeolite phase was the same as that in the experiments where the process time was 24 h (1A and 1B). By adding 1% LiCl to the experiments that ran at 100 °C for 24 h (1A and 1B), almost the same amount of the zeolite phase was obtained as that in the experiments that ran under the same temperature with a process time of 48 h and without 1% LiCl (sample No. 2). Thus, the addition of a 1% LiCl solution appears to be suitable for processes that run at 100 °C for 24 h where the yield of the zeolite phase is accelerated in a shorter time. Increasing the temperature to 120 °C (in the presence of 1% LiCl (2–4 mL) solution and a process time of 24 h) caused a small increase in the zeolite phase (~20%) compared to the sample without LiCl (sample No. 3 = 17.1%). An extension of the process time to 48 h (4A and 4B) had a positive effect on the amount of the zeolite phase, but it was not as significant as in the experiment with the same conditions but without 1% LiCl (sample No. 4 ≈ 23.7% of zeolite).

The effect of time and temperature with the addition of 10% LiCl is schematically illustrated in the blue part of Scheme 2. In the experiments where the temperature was 100 °C and the process time 48 h (4D), the content of the zeolite phase reached 27.5%. There was a significant difference in the content of the zeolite phase compared with that of the samples where 1% LiCl was used with the same temperature and process time of 24–48 h (1A, 1B, 1C, 1D, 2A, and 2B) or with that of the samples with the same temperature and different process times (24–48 h) but without LiCl (samples No. 1 and No. 2).

However, no difference or a small decrease in the zeolite phase content was visible in experiments where 10% LiCl was used with a temperature of 120 °C and a process time of 24–48 h (5E and 6F ≈ 25% of the sodalite phase only). Compared to samples A and 4B (containing 26–28% of the total zeolite phase), 1% of LiCl was more suitable than 10%.

In some cases, the addition of LiCl appears to be suitable for the process with a temperatures of 100 °C. With a longer process time (48 h) or a higher temperature (120 °C), it appears that time and temperature are more important factors than LiCl.

#### 4.1.3. Si/Al Ratio

The ratio between Si and Al was also studied by aqueous glass (as a source of Si) and Al_2_O_3_ additions. The ideal Si/Al ratio is around 2:1 to 1:1 [10,26,27]. In two experiments, the Si ratio of the component was increased from a Si/Al ratio of 2:1 (which contained the input coal fly ash) to a Si/Al ratio of 3:1 (see Table 4, samples 1C and 1D). This was done to confirm the theory that increasing the Si content leads to a decrease in the yield of the zeolite phase in the process of hydrothermal synthesis. The result from these experiments (Table 4, 1C and 1D) show that increasing Si/Al to 3:1 led to a radical reduction of the zeolite phase content compared to that of the other experiments, which were performed under the same conditions but with an Si/Al ratio of 2:1 (1A, 1B). Increasing the Si/Al ratio resulted in a decrease in the zeolite phase content of the product.

**Scheme 3:**
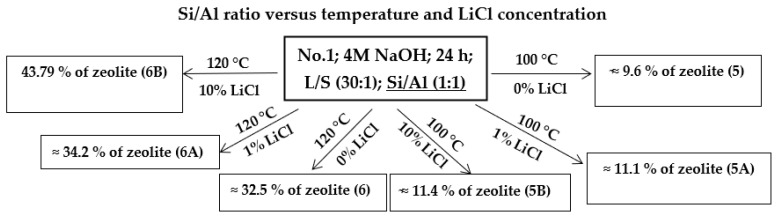
Hydrothermal synthesis of zeolites with Al_2_O_3_ (Si/Al ratio: 1:1): influence of temperature and LiCl.

In further experiments (Scheme 3), the Al content of the component was increased from an Si/Al ratio of 2:1 to a 1:1 ratio (samples 5, 5A, 5B, 6, 6A, and 6B), where Al_2_O_3_ was added to the input sample in such an amount that the final product contained a 1:1 ratio. The effect of Al_2_O_3_ was studied at two different temperatures (100 and 120 °C) and various concentrations of LiCl (1 and 10%). In samples where the experiments were performed at 100 °C and (samples 5, 5A, and 5B) a lower proportion of the zeolite phase can be seen in the products when compared to that of the same experiments without Al_2_O_3_ addition (1A and 1B). On the other hand, when we compare the samples without the addition of LiCl (sample No. 1 and No. 5), Al_2_O_3_ causes a significant increase in the zeolite phase formation (sample No. 5 contained 8.9% of SOD and 0.74% of FAU Na).

At a process temperature of 120 °C and a process length of 24 h, the samples with the Si/Al ratio changed to 1:1 (6, 6A and 6B) showed an increasing yield of the zeolite phase compared to parallel experiments that did not have the altered Al/Si ratio (3, 3B, and 5E). Compared to the original experiments without the addition of Al_2_O_3_ and LiCl (sample No. 3), the experiments with increased Al content (sample No. 6) contained a higher content of the sodalite phase (from 17.07 to 23.35%) as well as a higher content of phillipsite (from 0 to 9.22%). However, over time, as the LiCl catalyst concentration increased in the samples (6A and 6B), the phillipsite content decreased to zero (sample No. 6B), while the sodalite content increased (sample No. 6A: SOD—29.34%, PHI—4.90%; sample No. 6B: SOD—43.79%, PHI—0%) as the phillipsite decreased. These results show that changing the Si/Al ratio from 2:1 to 1:1 leads to an increased content of the sodalite phase in these experiments. These results are in accordance with results of other authors who confirmed that sodalite is formed prematurely in the process of hydrothermal synthesis at a Si/Al ratio 1:1 before other zeolite phases [12,14].

#### 4.1.4. Effect of Changing the Variable

**Scheme 4:**
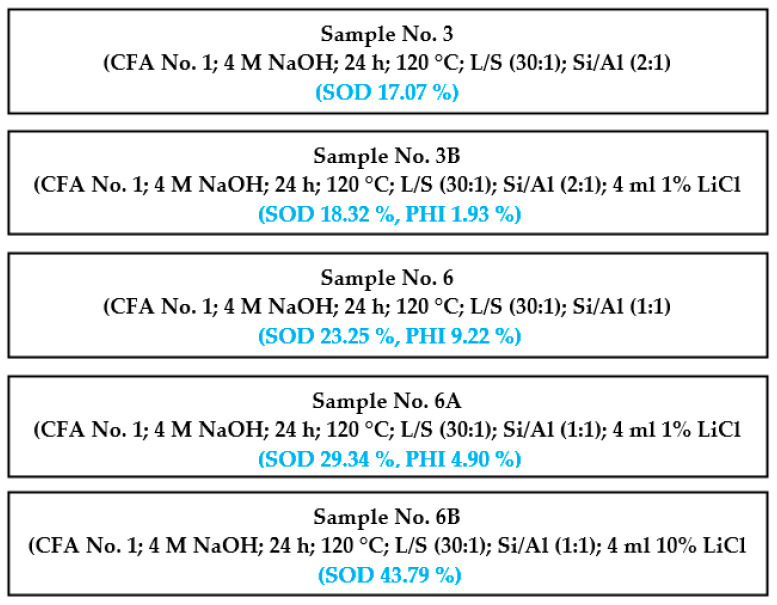
Effect of changing the variable.

In this section, the possibility of changing the variable for the purpose of obtaining a larger amount of the zeolite phase is shown step by step (samples No. 3, 3B, 6, 6A, and 6B). Scheme 4 shows one of the patterns that was used to produce more zeolite phases from knowledge that was acquired as we carried out our experiments.

Other patterns are not shown here but are visible in Table 4. Time, temperature, S/L ratio, and concentration of NaOH were the same for all of the experiments displayed in Scheme 4. Some parameters (Si/Al ratio and LiCl) were variable. It can be seen that the content of the zeolite phase increased in every process step when we changed a condition. The influence of the Si/Al ratio and concertation of LiCl on the process of hydrothermal synthesis is visible.

### 4.2. Phase Formation and Textural Properties during the Hydrothermal Process

**Figure 16:**
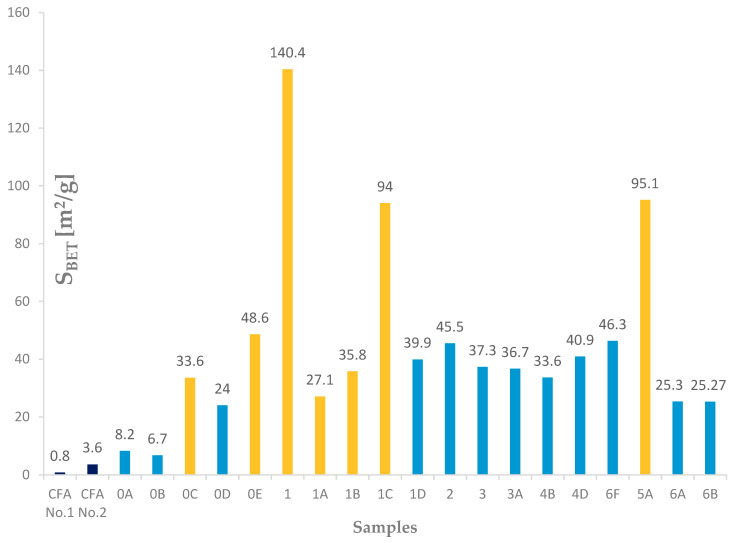
S_BET_ results for input fly ashes and experiments.

Different types and different amounts of zeolite phases formed during hydrothermal synthesis caused different textural properties (S_BET_, S_t_, and V_micro_ pores). These properties were observed in the selected samples (0A, 0B, 0C, 0D, 0E, 1, 1A, 1B, 1C, 1D, 2, 3, 3A, 4B, 4D, 6F, 5A, 6A, and 6B). The textural property results of the samples after the hydrothermal synthesis process were affected by the zeolite phase and also by the residues. Considering only the S_BET_ results, which were calculated for samples (3, 3A, 4D, 6F, 4B, 6A, and 6B) with a higher content of the zeolite phase (mostly sodalite and a minor amount of phillipsite) were between 23.27 and 46.3 m^2^/g. This is from 30 to 60 times more than that for the input coal fly ash No. 1, which had a S_BET_ value of 0.8 m^2^/g, as shown in Figure 16 and Table 4. Unlike these samples, the products that contained a smaller ratio of sodalite to Na (Ca-) FAU zeolite were mostly microporous. For these products, the calculated S_BET_ values were not of physical meaning, and other methods of characterization should be applied to determine their surface area, which should be much higher. It can be concluded that they correspond to the values measured for coal fly ash after completing the hydrothermal synthesis process where mostly sodalite was created, which are 33 and 43.6 m^2^/g [25].

In most experiments, there was an increased occurrence of the zeolite phase; there was also a complete or majority decomposition of mullite and quartz due to the composition of the original sample before hydrothermal synthesis. In general, the resultant product had a higher specific surface area, and the volume of the micropore increased when compared to the input samples.

## 5. Conclusions

In this work, coal fly ashes were hydrothermally treated to prepare synthetic zeolites. Zeolite phases, such as sodalite, phillipsite, chabazite, faujasite Na, and faujasite-Ca, were identified in almost all hydrothermal synthesis products. The content of these zeolite phases ranged from 2.09 to 43.79%.

Due to the factors that influenced the process of hydrothermal synthesis, it was concluded that the most significant factors that contributed to increasing the yield of the zeolite phase, also due to shortening the process time, were the following conditions in the following order: NaOH concentration = temperature, Al/Si ratio, and LiCl. The main conclusions from zeolitization experiments are as follows: 

(i) The zeolitization process was more successfully completed with coal fly ash No. 1 due to its better texture properties, lower grain size, and lower content of iron oxides

(ii) When temperature and time were the only variables (no LiCl or Al and Si addition), we can conclude that extending the process time positively influenced the hydrothermal process in the experiments at both temperatures (100 and 120 °C), although the positive effect was more visible at 100 °C.

(iii) The results from the addition of LiCl show that a low concentration of LiCl (1%) as well as a relatively high concentration (10%) did not affect the process as expected; however, it was noted that the zeolite phase was accelerated in a shorter time (see 24 h experiment).

(iv) Increasing/decreasing the Si/Al ratio significantly affected the yield of zeolites. A water glass (Si/Al ratio of 3:1) negatively influences the process. On the other hand, t the highest content of zeolite was achieved with the addition of Al_2_O_3_ (Si/Al ratio: 1:1) combined with LiCl (10%) and a temperature 120 °C (together with 4 M NaOH, an L/S ratio of 30:1, a process time of 24 h, and a crystallization phase for 16 h at 50 °C).

The measured data processed from the physical adsorption by the BET isotherm and t-plot method show that the values of the specific surface area, external surface, and the volume of micropores of the product are related to the values of the input coal fly ash sample. The S_BET_ values, which were calculated for the samples with a higher content of the zeolite phase (mostly sodalite and a small amount of phillipsite), were between 23.27 and 46.3 m^2^/g, which is from 30 to 60 times more than that for the input coal fly ash.

The results of these experiments show that a convection hydrothermal method can be effectively used to convert coal fly ash as CPPs into a zeolite phase, which may have better properties than input coal fly ash.

## Figures and Tables

**Table 1:** Overview of methods, process conditions, and formed zeolite phases.

Methods	Chemicals	L/S	T (°C)	t (h)	Zeolites	Reference
Hydrothermal method	NaOH, KOH, Na_2_CO_3_	8	90–150	24–96	Chabazite, Na-P1, phillipsite, sodalite, faujasite, zeolites (4A,A,P, X, Y)	[9,10,11,12,13]
Hydrothermal method using microwave waves	NaOH	8	100	0.25–2	Na-P1	[14,15,16]
Molten salt method	KOH, KNO_3_, NaOH, NaNO_3_ NH_4_F, NH_4_NO_3_	-	350	3–24	Sodalite, cancrinite	[17,18]
Fusion and hydrothermal method	NaOH, H_2_O, Na_3_AlO_3_	10	500–650	1–2	Faujasite, Na-A, Na-X, zeolite X	[9,18,19,20]

**Table 2:** Results of X-ray fluorescence (XRF) analysis of coal fly ashes No. 1 and No. 2.

XRF Phase (wt.%)	CFA No. 1	CFA No. 2	XRF Element (mg/kg)	CFA No. 1	CFA No. 2
**Fe_2_O_3_**	7.90	10.60	**V**	191	193
**Na_2_O**	0.59	0.39	**Cr**	166	169
**MgO**	1.93	2.01	**Ni**	122	113
**Al_2_O_3_**	23.3	20.3	**Cu**	134	124
**SiO_2_**	52.6	55.0	**Zn**	245	100
**P_2_O_5_**	0.18	0.11	**Rb**	154	177
**SO_3_**	0.69	0.21	**Sr**	321	271
**Cl**	0.01	0.00	**Zr**	188	326
**CaO**	4.02	5.45	**Ba**	1171	893
**TiO_2_**	1.07	1.20	**Pb**	131	43
**K_2_O**	3.52	3.67			
**LOI**	4.3	6.5			

**Table 3:** Content of mineral phase from XRD results with and without ZnO in coal fly ashes No. 1 and No. 2.

**Coal fly ash No. 1 (%)**								
**Mineral Phase**	**ZnO**	**Quartz**	**Mullite**	**Limestone**	**Periclase**	**Fe_2_O_3_**	**Fe_3_O_4_**	**Amorphous**
With ZnO	8.19	16.12	16.63	0.52	0.68	2.11	1	54.75
Without ZnO	-	17.56	18.11	0.57	0.74	2.30	1.09	59.63
**Coal fly ash No. 2 (%)**								
**Mineral Phase**	**ZnO**	**Quartz**	**Mullite**	**Limestone**	**Periclase**	**Fe_2_O_3_**	**Fe_3_O_4_**	**Amorphous**
With ZnO	7.61	30.43	8.96	-	-	0.78	0.82	51.40
Without ZnO	-	32.94	9.70	-	-	0.84	0.89	55.63

Amorphous phase calculated by difference (according to 100 mineral phases (%)).

**Table 4:** Experimental conditions of the hydrothermal process, textural properties, and amount of zeolite phases (blue-marked results have a negative C value in the Brunnauer–Emmett–Teller (BET) model. C. P.—crystallization phase (16 h, 50 °C); ULD—under limit of detection; Aq. g.—aqueous glass).

Sample	Coal Fly Ash	L/S	NaOH	Aq. g.	Al_2_O_3_	LiCl	T (°C)	t (h)	C. P.	S_BET_/S_t_ (m^2^/g)	V_micro_ (cm^3^/g)	Zeolite Phase
0	No. 2	7.5:1	2 M	-	-	-	90 °C	6 h	No	-	-	-
0A	No. 1	7.5:1	2 M	-	-	-	90 °C	6 h	No	S_BET_ 8.2/S_t_ ULD	ULD	-
0B	No. 2	7.5:1	2 M	-	-	-	110 °C	6 h	No	S_BET_ 6.7/S_t_ ULD	ULD	-
0C	No. 1	7.5:1	2 M	-	-	-	110 °C	6 h	No	S_BET_ 33.6/S_t_ 13.3	0.0091	CHA 4.11%, FAU Na 0.32%
0D	No. 2	7.5:1	3 M	-	-	-	110 °C	6 h	No	S_BET_ 24.0/S_t_ 19.0	0.0022	-
0E	No. 1	7.5:1	3 M	-	-	-	110 °C	6 h	No	S_BET_ 48.6/S_t_ 20.8	0.0128	CHA 4.68%, FAU Na 0.52%
1	No. 1	30:1	4 M	-	-	-	100 °C	24 h	Yes	S_BET_ 140.4/S_t_ 36.6	0.0459	SOD 0.68%, FAU Na 1.41%
1A	No. 1	30:1	4 M	-	-	2 mL 1%	100 °C	24 h	Yes	S_BET_ 27.1/S_t_ 26.2	0.0109	SOD 15.64%
1B	No. 1	30:1	4 M	-	-	4 mL 1%	100 °C	24 h	Yes	S_BET_ 35.8/S_t_ 23.8	0.0054	SOD 15.61%, FAU Na 0.18%
1C	No. 1	30:1	4 M	10 mL	-	2 mL 1%	100 °C	24 h	Yes	S_BET_ 94.0/S_t_ 37.6	0.262	SOD 7.14%, FAU Ca 0.64%
1D	No. 1	30:1	4 M	10 mL	-	4 mL 1%	100 °C	24 h	Yes	S_BET_ 39.9/S_t_ 30.1	0.0043	SOD 2.73%, PHI 7.33%
2	No. 1	30:1	4 M	-	-	-	100 °C	48 h	Yes	S_BET_ 45.5/S_t_ 44.2	ULD	SOD 14.54%
2A	No. 1	30:1	4 M	-	-	2 mL 1%	100 °C	48 h	Yes	-	-	SOD 15.57%, FAU Na 0.29%
2B	No. 1	30:1	4 M	-	-	4 mL 1%	100 °C	48 h	Yes	-	-	SOD 14.54%
3	No. 1	30:1	4 M	-	-	-	120 °C	24 h	Yes	S_BET_ 37.3/S_t_ 28.0	0.040	SOD 17.07%
3A	No. 1	30:1	4 M	-	-	2 mL 1%	120 °C	24 h	Yes	S_BET_ 36.7/S_t_ 25.8	0.0046	SOD 18.74%, PHI 1.88%
3B	No. 1	30:1	4 M	-	-	4 mL 1%	120 °C	24 h	Yes	-	-	SOD 18.32%, PHI 1.93%
4	No. 1	30:1	4 M	-	-	-	120 °C	48 h	Yes	-	-	SOD 23.72%
4A	No. 1	30:1	4 M	-	-	2 mL 1%	120 °C	48 h	Yes	-	-	SOD 23.68%, PHI 2.28%
4B	No. 1	30:1	4 M	-	-	4 mL 1%	120 °C	48 h	Yes	S_BET_ 33.6/S_t_ 23.5	0.0044	SOD 26.37%, PHI 2.10%
4C	No. 1	30:1	4 M	-	-	2 mL 10%	100 °C	24 h	Yes	-	-	SOD 10.68%, FAU Na 0.86%
4D	No. 1	30:1	4 M	-	-	2 mL 10%	100 °C	48 h	Yes	S_BET_ 40.9/S_t_ 30.1	0.0050	SOD 27.48%
5E	No. 1	30:1	4 M	-	-	2 mL 10%	120 °C	24 h	Yes	-	-	SOD 24.85%
6F	No. 1	30:1	4 M	-	-	2 mL 10%	120 °C	48 h	Yes	S_BET_ 46.3/S_t_ 35.6	0.0045	SOD 25.12%
5	No. 1	30:1	4 M	-	1.1558 g	-	100 °C	24 h	Yes	-	-	SOD 8.90%, FAU Na 0.74%
5A	No. 1	30:1	4 M	-	1.1558 g	4 mL 1%	100 °C	24 h	Yes	S_BET_ 95.1/S_t_ 51.5	0.0198	SOD 10.46%, FAU Na 0.68%
5B	No. 1	30:1	4 M	-	1.1558 g	4 mL 10%	100 °C	24 h	Yes	-	-	SOD 10.72%, FAU Na 0.67%
6	No. 1	30:1	4 M	-	1.1558 g	-	120 °C	24 h	Yes	-	-	SOD 23.35%, PHI 9.22%
6A	No. 1	30:1	4 M	-	1.1558 g	4 mL 1%	120 °C	24 h	Yes	S_BET_ 25.3/S_t_ 21.0	0.0018	SOD 29.34%, PHI 4.90%
6B	No. 1	30:1	4 M	-	1.1558 g	4 mL 10%	120 °C	24 h	Yes	S_BET_ 23.27/S_t_ 17.6	-	SOD 43.79%

**Table 5:** XRD results for samples No. 4A 6 and 6B and the input—coal fly ash No. 1 (%).

Sample/Mineral Phase	ZnO	Quartz	Mullite	Sodalite	Phillipsite	Amorphous
Sample No.4A with ZnO	10.90	0.71	4.79	21.10	2.03	60.48
Sample No. 4A without ZnO	-	0.80	5.38	23.68	2.28	67.88
Sample No. 6 with ZnO	9.09	3.05	-	21.23	8.38	58.25
Sample No. 6 without ZnO	-	3.36	-	23.35	9.22	64.07
Sample No. 6B with ZnO	9.09	1.60	1.22	39.81	-	48.28
Sample No. 6B without ZnO	-	1.76	1.34	43.79	-	53.11
CFA No. 1 without ZnO	-	17.76	18.11	-	-	59.63

## Data Availability

The data presented in this study are available in Längauer, D.; Čablík, V.; Hredzák, S.; Zubrik, A.; Matik, M.; Danková, Z. Preparation of Synthetic Zeolites from Coal Fly Ash by Hydrothermal Synthesis. *Materials* **2021**, *14*, 1267. https://doi.org/10.3390/ma14051267 (accessed on 7 March 2021).

## Abbreviations

CCPs	Coal combustion products
CFA	Coal fly ash
XRF	X-ray fluorescence
SEM	Scanning electron microscopy
XRD	X-ray diffraction
EDX	Energy Dispersive X-Ray Spectroscopy
LOI	Loss on ignition
SOD	Sodalite
PHI	Phillipsite
FAU	Faujasite
CHA	Chabazite
S_t_	External surface area
S_BET_	Specific surface area
V_micro_	Volume of micropore

## References

[1] Harris D., Heidrich C., Feuerborn J., Global Aspects on Coal Combustion Products (2019). Coaltrans. https://www.coaltrans.com/insights/article/global-aspects-on-coal-combustion-products.

[2] European Coal Combustion Products Association e.V. ECOBA Statistics 2016. Essen: ECOBA e.V.. http://www.ecoba.com/.

[3] Ryu G.U., Khalid H.R., Lee N., Wang Z., Lee H.K. (2021). The Effects of NaOH Concentration on the Hydrothermal Synthesis of a Hydroxyapatite–Zeolite Composite Using Blast Furnace Slag. Minerals.

[4] Kang Y., Swain B., Im B., Yoon J.-H., Park K.H., Lee C.G., Kim D.G. (2019). Synthesis of Zeolite Using Aluminum Dross and Waste LCD Glass Powder: A Waste to Waste Integration Valorization Process. Metals.

[5] Łach M., Grela A., Komar N., Mikuła J., Hebda M. (2019). Calcined Post-Production Waste as Materials Suitable for the Hydrothermal Synthesis of Zeolites. Materials.

[6] Jha B., Singh D.N. (2016). Fly Ash Zeolites.

[7] Chindaprasirt P., Rattanasak U. (2009). Utilization of blended fluidized bed combustion (FBC) ash and pulverized coal combustion (PCC) fly ash in geopolymer. Waste Manag..

[8] Jiang Z., Yang J., Ma H., Ma X., Yuan J. (2016). Synthesis of pure NaA zeolites from coal fly ashes for ammonium removal from aqueous solutions. Clean Technol. Environ. Policy.

[9] Rayalu S., Meshram S.U., Hasan M.Z. (2000). Highly crystalline faujasitic zeolites from flyash. J. Hazard. Mater..

[10] Adamczyk Z., Białecka B. (2005). Hydrothermal Synthesis of Zeolites from Polish Coal Fly Ash. Polish J. Environ. Stud..

[11] Murayama N., Yamamoto H., Shibata J. (2002). Zeolite synthesis from coal fly ash by hydrothermal reaction using various alkali sources. J. Chem. Technol. Biotechnol..

[12] Lee N.K., Khalid H.R., Lee H.K. (2016). Synthesis of mesoporous geopolymers containing zeolite phases by a hydrothermal treatment. Microporous Mesoporous Mater..

[13] Khalid H.R., Lee N.K., Park S.M., Abbas N., Lee H.K. (2018). Synthesis of geopolymer-supported zeolites via robust one-step method and their adsorption potential. J. Hazard. Mater..

[14] Inada M., Eguchi Y., Enomoto N., Hojo J. (2005). Synthesis of zeolite from coal fly ashes with different silica–alumina composition. Fuel.

[15] Tanaka H., Eguchi H., Fujimoto S., Hino R. (2006). Two-step process for synthesis of a single phase Na–A zeolite from coal fly ash by dialysis. Fuel.

[16] Fukui K., Katoh M., Yamamoto T., Yoshida H. (2009). Utilization of NaCl for phillipsite synthesis from fly ash by hydrothermal treatment with microwave heating. Adv. Powder Technol..

[17] Park M., Choi C.L., Lim W.T., Kim M.C., Choi J., Heo N.H. (2000). Molten-salt method for the synthesis of zeolitic materials I. Zeolite formation in alkaline molten-salt system. Microporous Mesoporous Mater..

[18] Park M., Choi C.L., Lim W.T., Kim M.C., Choi J., Heo N.H. (2000). Molten-salt method for the synthesis of zeolitic materials II. Characterization of zeolitic materials. Microporous Mesoporous Mater..

[19] Berkgaut V., Singer A. (1996). High capacity cation exchanger by hydrothermal zeolitization of coal fly ash. Appl. Clay Sci..

[20] Thomson K.T. (2004). Handbook of Zeolite Science and Technology; Auerbach, S.M., Carrado, K.A., Dutta, P.K., Eds.; Marcel Dekker, Inc.: New York, NY, USA; Marcel Dekker, Inc.: Basel., Switzerland, 2003. xii. J. Am. Chem. Soc..

[21] Somerset V., Petrik L., Iwuoha E. (2008). Alkaline hydrothermal conversion of fly ash precipitates into zeolites 3: The removal of mercury and lead ions from wastewater. J. Environ. Manag..

[22] Byoung J.A. (1997). Synthesis of Na-P1 Zeolite from Coal Fly Ash. J. Korean Soc. Atmos. Environ..

[23] Fernández-Pereira C., Galiano Y.L., Rodríguez-Piñero M.A., Vale J., Querol X. (2002). Utilisation of zeolitised coal fly ash as immobilising agent of a metallurgical waste. J. Chem. Technol. Biotechnol..

[24] Hollman G., Steenbruggen G., Janssen-Jurkovičová M. (1999). A two-step process for the synthesis of zeolites from coal fly ash. Fuel.

[25] Franus W., Wdowin M., Franus M. (2014). Synthesis and characterization of zeolites prepared from industrial fly ash. Environ. Monit. Assess..

[26] Wałek T.T., Saito F., Zhang Q. (2008). The effect of low solid/liquid ratio on hydrothermal synthesis of zeolites from fly ash. Fuel.

[27] Wdowin M., Franus M., Panek R., Badura L., Franus W. (2014). The conversion technology of fly ash into zeolites. Clean Technol. Environ. Policy.

[28] Pedrolo D.R.S., de Menezes Quines L.K., de Souza G., Marcilio N.R. (2017). Synthesis of zeolites from Brazilian coal ash and its application in SO_2_ adsorption. J. Environ. Chem. Eng..

[29] Inada M., Tsujimoto H., Eguchi Y., Enomoto N., Hojo J. (2005). Microwave-assisted zeolite synthesis from coal fly ash in hydrothermal process. Fuel.

[30] Esaifan M., Warr L.N., Grathoff G., Meyer T., Schafmeister M.-T., Kruth A., Testrich H. (2019). Synthesis of Hydroxy-Sodalite/Cancrinite Zeolites from Calcite-Bearing Kaolin for the Removal of Heavy Metal Ions in Aqueous Media. Minerals.

[31] Skupina CEZ Elektrárna Dětmarovice. https://www.cez.cz.

[32] Brunauer S., Emmett P.H., Teller E. (1938). Adsorption of Gases in Multimolecular Layers. J. Am. Chem. Soc..

[33] Hudec P., Novanský J., Silhár S., Trung T.N., Zúbek M., Maďar J. (1986). Possibility of Using t-Plots, Obtained from Nitrogen Adsorption for the Valuation of Zeolites. Adsorpt. Sci. Technol..

[34] Panitchakarn P., Laosiripojana N., Viriya-umpikul N., Pavasant P. (2014). Synthesis of high-purity Na-A and Na-X zeolite from coal fly ash. J. Air Waste Manag. Assoc..

[35] Sing K.S.W. (1982). Reporting physisorption data for gas/solid systems with special reference to the determination of surface area and porosity (Provisional). Pure Appl. Chem..

[36] Sing K.S.W., Williams R.T. (2005). Empirical Procedures for the Analysis of Physisorption Isotherms. Adsorpt. Sci. Technol..

[37] Smith J.V. (1963). Structural Classification of Zeolites.

[38] Vávra V. (2013). Atlas Minerálů: Silikáty—Tektosilikáty—Skupina Zeolitů.

[39] Oh J.E., Moon J., Mancio M., Clark S.M., Monteiro P.J.M. (2011). Bulk modulus of basic sodalite, Na_8_ [AlSiO_4_] 6 (OH)_2_·2H_2_O, a possible zeolitic precursor in coal-fly-ash-based geopolymers. Cem. Concr. Res..

[40] Feng W., Wan Z., Daniels J., Li Z., Xiao G., Yu J., Xu D., Guo H., Zhang D., May E.F. (2018). Synthesis of high quality zeolites from coal fly ash: Mobility of hazardous elements and environmental applications. J. Clean. Prod..

[41] Belviso C. (2018). Ultrasonic vs. hydrothermal method: Different approaches to convert fly ash into zeolite. How they affect the stability of synthetic products over time?. Ultrason. Sonochem..

[42] Yao Z.T., Xia M.S., Ye Y., Zhang L. (2009). Synthesis of zeolite Li-ABW from fly ash by fusion method. J. Hazard. Mater..

